# β-Asarone Alleviates High-Glucose-Induced Oxidative Damage via Inhibition of ROS Generation and Inactivation of the NF-κB/NLRP3 Inflammasome Pathway in Human Retinal Pigment Epithelial Cells

**DOI:** 10.3390/antiox12071410

**Published:** 2023-07-11

**Authors:** Cheol Park, Hee-Jae Cha, Hyun Hwangbo, EunJin Bang, Su Hyun Hong, Kyoung Seob Song, Jeong Sook Noh, Do-Hyung Kim, Gi-Young Kim, Yung Hyun Choi

**Affiliations:** 1 Department Division of Basic Sciences, College of Liberal Studies, Dong-eui University, Busan 47340, Republic of Korea; 2 Department of Parasitology and Genetics, College of Medicine, Kosin University, Busan 49104, Republic of Korea; 3 Anti-Aging Research Center, Dong-eui University, Busan 47340, Republic of Korea; 4 Department of Biochemistry, College of Korean Medicine, Dong-eui University, Busan 47340, Republic of Korea; 5 Department of Medical Life Science, College of Medicine, Kosin University, Busan 49104, Republic of Korea; 6 Department of Food Science & Nutrition, Tongmyong University, Busan 48520, Republic of Korea; 7 Department of Aquatic Life Medicine, College of Fisheries Sciences, Pukyong National University, Busan 48513, Republic of Korea; 8 Department of Marine Life Science, Jeju National University, Jeju 63243, Republic of Korea

**Keywords:** β-asarone, high glucose, ROS, NF-κB, NLRP3 inflammasome

## Abstract

Diabetic retinopathy (DR) is the leading cause of vision loss and a major complication of diabetes. Hyperglycemia-induced accumulation of reactive oxygen species (ROS) is an important risk factor for DR. β-asarone, a major component of volatile oil extracted from *Acori graminei* Rhizoma, exerts antioxidant effects; however, its efficacy in DR remains unknown. In this study, we investigated whether β-asarone inhibits high-glucose (HG)-induced oxidative damage in human retinal pigment epithelial (RPE) ARPE-19 cells. We found that β-asarone significantly alleviated cytotoxicity, apoptosis, and DNA damage in HG-treated ARPE-19 cells via scavenging of ROS generation. β-Asarone also significantly attenuated the excessive accumulation of lactate dehydrogenase and mitochondrial ROS by increasing the manganese superoxide dismutase and glutathione activities. HG conditions markedly increased the release of interleukin (IL)-1β and IL-18 and upregulated their protein expression and activation of the nuclear factor-kappa B (NF-κB) signaling pathway, whereas β-asarone reversed these effects. Moreover, expression levels of the NOD-like receptor family pyrin domain-containing 3 (NLRP3) inflammasome multiprotein complex molecules, including thioredoxin-interacting protein, NLRP3, apoptosis-associated speck-like protein containing a caspase-recruitment domain, and cysteinyl aspartate-specific proteinase-1, were increased in ARPE-19 cells under HG conditions. However, their expression levels remained similar to those in the control group in the presence of β-asarone. Therefore, β-asarone protects RPE cells from HG-induced injury by blocking ROS generation and NF-κB/NLRP3 inflammasome activation, indicating its potential as a therapeutic agent for DR treatment.

## 1. Introduction

Diabetic retinopathy (DR), the most common and severe complication of diabetes, is the major cause of visual impairment and vision loss. Hyperglycemia plays a crucial role in DR pathogenesis [1,2]. Oxidative and inflammatory stress induced by hyperglycemia are involved in the initiation and progression of DR. Accumulation of ROS promote increased oxidative stress due to hyperglycemia, is a key factor in DR-associated retinal pigment epithelial (RPE) cell damage [3,4,5]. Elevated intracellular ROS levels trigger inflammation and lead to the occurrence and progression of chronic diseases, such as DR. Mitochondria are the major site of ROS generation, which directly stimulate pro-inflammatory cytokine secretion and promote pathological conditions [6]. Hyperglycemia increases the mitochondrial ROS levels, resulting in a chronic inflammatory state [7,8]. Therefore, alleviation of oxidative stress and inflammation may be a promising strategy for the treatment of DR.

Recent studies have suggested the involvement of the NLR family pyrin domain-containing-3 (NLRP3) inflammasome signaling pathway in DR pathogenesis [9,10,11]. Abnormal activation of this signaling pathway is closely related to aging-related diseases, such as diabetes. Expression levels of the NLRP3 inflammasome multiprotein complex molecules, apoptosis-associated speck-like protein containing a caspase-recruitment domain (ASC) and caspase-1, are markedly increased in patients with DR [12,13,14]. Activation of the NLRP3 inflammasome pathway is initiated by various stimuli, such as pathogenic molecules, mitochondrial damage, and excessive ROS accumulation. This pathway is divided into canonical and non-canonical pathways depending on the activation of cysteine aspartase during inflammasome formation [15,16]. In canonical signaling, NLRP3 inflammasome priming leads to the nuclear translocation of nuclear factor-kappa B (NF-κB), promoting the expression of precursor proteins, including NLRP3, ASC, pro-caspase-1, pro-interleukin (IL)-1β, and pro-IL-18, which are essential mediators of inflammation. However, in non-canonical signaling pathways, NLRP3 becomes activated through cleaved caspase-11 [17]. Therefore, inflammasome inactivation may be a novel therapeutic strategy to ameliorate the symptoms of various inflammatory diseases, including DR [12,18].

β-Asarone (*cis*-2,4,5-trimethoxy-1-allyl phenyl) is a volatile oil present in various herbal medicines, including *Acorus tatarinowii* Schott. It exerts various biological effects, including antioxidant, anti-inflammatory, and anti-cancer effects [19,20]. For example, β-asarone suppresses the production of pro-inflammatory mediators and cytokines by blocking the NF-κB signaling pathway and preventing the development of neuroinflammatory diseases [21]. In addition, β-asarone directly scavenges hydrogen peroxide and hydroxyl radicals and exerts protective effects against DNA oxidation [22]. Recently, Cai et al. [23] reported that a decrease in amyloid-β-induced neuronal apoptosis and improvement in learning and memory by β-asarone were closely related to the reduction in oxidative stress. Xiao et al. [24] revealed that β-asarone mitigates myocardial ischemia/reperfusion (I/R) injury via the inhibition of NLRP3 inflammasome-mediated lytic cell death. However, the protective effects of β-asarone against oxidative stress and inflammation in hyperglycemic RPE cells and its action mechanism remain unknown. To the best of our knowledge, this study is the first to examine the effects of β-asarone on ROS-mediated NF-κB/NLRP3 inflammasome activation in high-glucose (HG)-induced hyperglycemic RPE cells.

## 2. Materials and Methods

### 2.1. Cell Culture and Treatment

ARPE-19 cells (CRL-2302; American Type Culture Collection, Manassas, VA, USA) were cultured as previously described [25]. β-Asarone was obtained from Sigma-Aldrich Co. (St. Louis, MO, USA), and the materials necessary for cell culture were provided by WelGENE Inc. (Gyungsan, Korea). Cells were grown for 48 h in a medium with various doses of β-asarone (0, 10, 25, 50, and 100 μM) and glucose (0, 5, 10, 20, and 40 mM) or pre-treated with optimal concentration of β-asarone (50 μM) or N-acetyl-cysteine (NAC; 10 mM; Thermo Fisher Scientific, Waltham, MA, USA) for 1 h and cultured for an additional 48 h under HG (20 mM) conditions in presence of β-asarone and NAC. The optimal treatment concentrations for β-asarone (50 μM) were selected, as was the highest treatment concentration that showed no decreased cell viability in ARPE-19 cells. The optimal concentration for glucose (20 mM) was selected, as was the lowest treatment concentration that showed sublethal levels of cell viability.

### 2.2. Cell Viability and Lactate Dehydrogenase (LDH) Assays

After treatment, cell viability was examined using the 3-(4,5-dimethyl-2-thiazolyl)-2,5-diphenyltetrazolium bromide (MTT) assay (Sigma-Aldrich Co., St. Louis, MO, USA), as previously described [26]. The level of LDH released from the cells into the culture medium was determined using an LDH Cytotoxicity Assay Kit (Thermo Fisher Scientific, Waltham, MA, USA), according to the manufacturer’s instructions. To measure LDH activity, the absorbance was measured at 490 nm and 680 nm, in which an absorbance value of 680 nm, a background signal, was subtracted from an absorbance value of 490 nm. The results are presented as values normalized to the control value.

### 2.3. Determination of the Cell and Nuclear Morphology

Morphological changes in cells treated with HG for 48 h with or without β-asarone (50 μM) were observed under an inverted microscope (Carl Zeiss, Oberkochen, Germany). The morphological changes due to cellular apoptosis in ARPE-19 cells are majorly characterized by disruption of cell monolayer and reduced cell volume, which could be observed under an inverted microscope. To assess the morphological changes in the nucleus, cells were stained with 4′,6-diamidino-2-phenylindole (DAPI; Thermo Fisher Scientific, Waltham, MA, USA) and examined under a fluorescence microscope (Carl Zeiss, Oberkochen, Germany) at the Core-Facility Center for Tissue Regeneration, Dong-eui University (Busan, Korea). The DAPI is a blue fluorescence dye, which could more easily enters the apoptotic cell and stains a stronger blue color. The changes in the nucleus morphology of apoptotic cells include chromosome condensation and fragmentation allows identification of apoptotic cells [27].

### 2.4. Apoptosis Assay

After culturing the cells under HG conditions in the presence or absence of β-asarone, apoptosis was detected via flow cytometry (Becton Dickinson, San Jose, CA, USA) using the fluorescein isothiocyanate (FITC) Annexin V Apoptosis Detection Kit (BD Biosciences, San Jose, CA, USA). To interpret the results, the percentages of early and late apoptotic cells (Annexin-V^+^/propidium iodide (PI)^−^ and Annexin-V^+^/PI^+^) were used to determine the total number of apoptotic cells [27].

### 2.5. Western Blot Analysis

Cells seeded at 2 × 10^5^ cells per well in 6-well plate were lysed using the radioimmunoprecipitation assay lysis buffer (Sigma-Aldrich Co., St. Louis, MO, USA) or NE-PER Nuclear and Cytoplasmic Extraction Reagent (Thermo Fisher Scientific, Waltham, MA, USA), according to the manufacturers’ protocols. The experiment was performed at least three times. Briefly, Cytoplasmic Extraction Reagent (CER) I reagent (100–200 μL) was added to cell pellet, followed by CER II reagent (5.5–11 μL). After short ice incubation, cells were centrifuged at 13,000× *g*, 4 °C for 10 min, which then collected supernatant or cytoplasmic protein extract was obtained. Pellet was suspended in ice-cold Nuclear Extraction Reagent. After ice incubation, the resuspended pellet was centrifuged at 13,000× *g*, 4 °C for 10 min. The collected supernatant fraction was obtained nuclear protein extract. The protein quantification was measured by using bicinchoninic acid (BCA) assay, in which proteins induce the reduction of alkaline Cu(II) to Cu(I) with increasing concentration, which BCA specifically forms complex with Cu(I), in which the complex exhibits purple color that can be detected by measuring absorbance at 562 nm. After protein quantification using the BCA Protein Assay Kit (Sigma-Aldrich Co., St. Louis, MO, USA), Western blot analysis was performed as previously described [28]. Antibodies were obtained from Santa Cruz Biotechnology, Inc. (Dallas, TX, USA), Cell Signaling Technology, Inc. (Beverly, MA, USA), Thermo Fisher Scientific, and Abcam (Cambridge, UK). Protein bands were detected using enhanced chemiluminescence reagents (Thermo Fisher Scientific, Waltham, MA, USA), following the manufacturer’s instructions.

### 2.6. Assessment of DNA Damage

To evaluate the effect of β-asarone on HG-induced oxidative DNA damage, the levels of 8-hydroxy-2′-deoxyguanosine (8-OHdG) in cells were determined using the 8-OHdG enzyme-linked immunosorbent assay (ELISA) kit (Abcam, Cambridge, UK), according to the manufacturer’s protocol. The concentrations of 8-OHdG obtained from the standard curve were expressed in ng/mL. In addition, single-stranded DNA breaks were detected to visualize the DNA damage using the Comet Assay kit (Trevigen, Gaithersburg, MD, USA), according to the manufacturer’s instructions. Comet images were examined using fluorescence microscopy.

### 2.7. ROS Assay

Cytosolic ROS levels were assessed using the 5,6-carboxy-2′,7′-dichlorodihydrofluorescein diacetate (DCF-DA) fluorescent probe (Thermo Fisher Scientific, Waltham, MA, USA). Briefly, cells treated with HG for 1 h with or without pre-treatment with β-asarone (50 μM) were collected and reacted with DCF-DA (20 μM), as per the manufacturer’s recommendation. After washing the cells with phosphate-buffered saline (PBS), the fluorescence intensity was measured using flow cytometry, and fluorescence images were acquired via fluorescence microscopy [29]. Similarly, mitochondrial ROS production was measured using the MitoSox red dye (Thermo Fisher Scientific, Waltham, MA, USA), which detects the level of superoxide anions in the mitochondria. At the end of treatment, the cells were stained with MitoSox (10 μM), and the fluorescence intensity was analyzed via flow cytometry and fluorescence microscopy [30].

### 2.8. Evaluation of the Antioxidant Defense System

At the end of treatment, the reduced glutathione (GSH)/oxidized glutathione (GSSG) ratio (GSH/GSSG Ratio Detection Assay Kit, Abcam, Cambridge, UK) and the activity of manganese superoxide dismutase (MnSOD; MnSOD Assay Kit, Cayman Chemical, Ann Arbor, MI, USA) in cells were measured using spectrophotometric and enzymatic methods with commercially available kits, according to the manufacturers’ instructions.

### 2.9. Measurement of IL-1β and IL-18 Levels

Following different treatments, cell supernatants were collected and the concentrations of IL-1β and IL-18 were quantified using specific ELISA kits (R&D Systems Inc., Minneapolis, MN, USA), following the manufacturer’s protocol. The absorbance of the final product was measured at 450 nm using a microplate reader (Beckman Coulter Inc., Brea, CA, USA), according to the method described by Hwangbo et al. [31].

### 2.10. Immunofluorescence Assay for NF-κB, NLRP3, and ASC

To determine the expression level of phosphorylated (p)-NF-κB via immunofluorescence staining, the cells were cultured for 1 h under HG conditions with or without β-asarone, and the expression levels of NLRP3 and ASC were determined after 48 h of culture. Briefly, cells were fixed with formaldehyde after treatment, permeabilized with Triton X-100, reacted with primary antibodies against p-NF-κB (Cell Signaling Technology Inc., Beverly, MA, USA), NLRP3 (Abcam, Cambridge, UK), and ASC (AdipoGen Life Sciences, San Diego, CA, USA), and incubated with FITC-tagged secondary antibody IgG and Alexa Fluor 594-tagged secondary antibody IgG (Thermo Fisher Scientific, Waltham, MA, USA). Subsequently, the nuclei were counterstained with DAPI, and fluorescence images were captured [32].

### 2.11. Assay of Caspase-1 Activity

Caspase-1 activity in HG-stimulated cells with or without β-asarone was detected using a colorimetric assay kit (R&D Systems Inc., Brea, CA, USA). After the cell pellets were lysed with the lysis buffer provided with the kit, the lysates were reacted with *p*-nitroanilide (pNA)-labeled substrates in the reaction buffer. The enzymatic activity of caspase-1 was calculated from a standard curve prepared using pNA, according to the manufacturer’s instructions.

### 2.12. Statistical Analysis

Data were analyzed via one-way analysis of variance with Tukey’s post hoc test using the GraphPad Prism software (GraphPad Software Inc., La Jolla, CA, USA). Statistical significance was set at *p* < 0.05.

## 3. Results

### 3.1. β-Asarone Ameliorates HG-Induced Cell Viability Reduction and Cytotoxicity in ARPE-19 Cells

**Figure 1:**
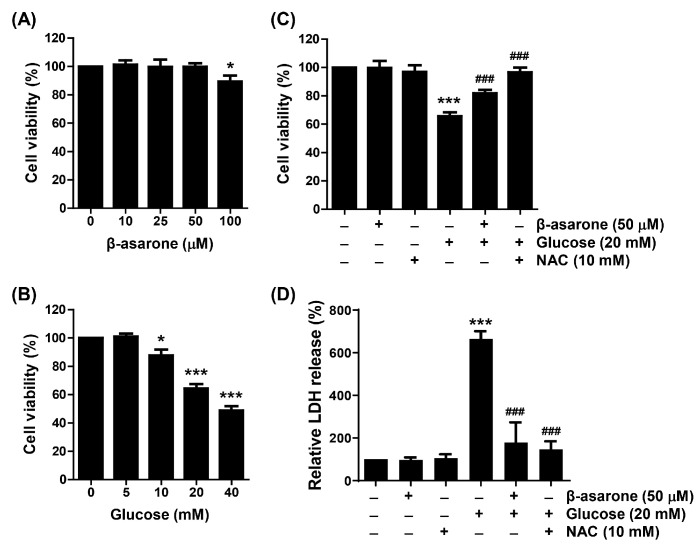
Inhibitory effects of β-asarone on high-glucose (HG)-induced cell viability reduction and cytotoxicity in ARPE-19 cells. Cells were grown for 48 h in a medium with various doses of β-asarone (0, 10, 25, 50, and 100 μM) (**A**) and glucose (0, 5, 10, 20, and 40 μM) (**B**) or pre-treated with β-asarone (50 μM) or *N*-acetyl-cysteine (NAC; 10 mM) for 1 h and cultured for an additional 48 h under HG (20 mM glucose) conditions in presence of β-asarone or NAC (**C**,**D**). Cell viability and cytotoxicity were determined using the 3-(4,5-dimethyl-2-thiazolyl)-2,5-diphenyltetrazolium bromide (MTT) (**A**–**C**) and lactate dehydrogenase (LDH) (**D**) assays, respectively. Numerical values are represented as the mean ± standard deviation of three independent experiments (SD; * *p* < 0.05 and *** *p* < 0.001 vs. control group; ### *p* < 0.001 vs. HG-treated group).

MTT assay was performed to examine the effect of β-asarone on the proliferation of ARPE-19 cells cultured under HG conditions. Compared to the control group, β-asarone did not show a notable difference in cell viability at concentrations <50 μM, but was slightly decreased in cells treated with 100 μM (Figure 1A). In cells treated with glucose, cell viability was significantly inhibited in a concentration-dependent manner. Notably, in cells treated with 20 mM glucose, viability decreased to approximately 60% (Figure 1B). Based on this result, the 20 mM glucose-treated group was set as the HG group, and the pre-treatment concentration of β-asarone was set as 50 μM. As shown in Figure 1C results, pre-treatment with 50 μM β-asarone significantly suppressed the inhibitory effect of HG on ARPE-19 cell viability, and the HG-induced decrease in cell viability was also reversed in NAC pre-treated cells. LDH assay revealed that β-asarone and NAC significantly ameliorated lytic cell death measured by LDH release HG-induced cytotoxicity in cells (Figure 1D).

### 3.2. β-Asarone Reduces Apoptosis and DNA Damage in HG-Treated ARPE-19 Cells

**Figure 2:**
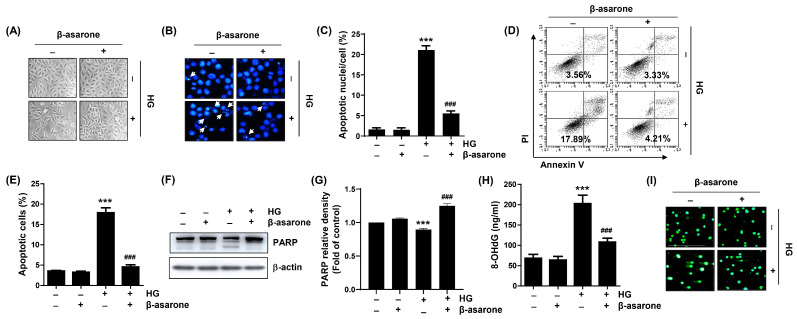
Inhibition of HG-induced apoptosis and DNA damage by β-asarone in ARPE-19 cells. Cells were treated with β-asarone (50 μM) for 1 h and cultured for an additional 48 h under HG (20 mM glucose) conditions. (**A**) Representative images of cell morphology changes as observed by using inverted microscope. (**B**,**C**) 4′,6-diamidino-2-phenylindole (DAPI) staining was performed to observe morphological changes in the nuclei by using fluorescence microscopy. Representative images of DAPI-stained nuclei (**B**) and quantification results of apoptotic nuclei (**C**). (**D**,**E**) Apoptosis was measured using flow cytometry after Annexin V-fluorescein isothiocyanate (FITC)/propidium iodide (PI) staining. Representative images of flow cytometry (**D**) and quantification results (**E**). (**F**,**G**) After the lysis of collected cells, equal amounts of protein from each cell lysate were loaded and blotted with poly(ADP ribose) polymerase (PARP) and β-actin antibodies. (**H**,**I**) DNA damage was assessed using the 8-hydro’y-2′-deoxyguanosine (8-OHdG) (**H**) and comet (**I**) assays. (**C**,**E**,**G**) Numerical values are represented as the mean ± SD of three independent experiments (*** *p* < 0.001 vs. control group; ### *p* < 0.001 vs. HG-treated group).

As indicated in Figure 2A, treatment of cells with HG induced changes in their cell morphology, such as rounding of cells and loss of cell anchorage to the culture plate, but these effects were not observed in β-asarone-pre-treated cells. Therefore, we evaluated whether the HG-induced inhibition of cell viability and morphological changes are associated with the induction of apoptosis and whether β-asarone suppresses apoptosis. DAPI staining and flow cytometry revealed that cells cultured under HG conditions showed a significant increase in apoptosis. However, the upward trend in the HG-mediated apoptosis rate was notably attenuated in the presence of β-asarone (Figure 2B–E). In addition, the cleavage of poly(ADP ribose) polymerase, a marker of apoptosis [27], in HG-treated cells was attenuated by pre-treatment with β-asarone (Figure 2F,G). Furthermore, the level of 8-OHdG, a marker of oxidative DNA damage [33], was significantly higher in HG-stimulated cells than in the control cells (Figure 2H). Furthermore, increased comet tail formation, which indicates broken DNA strands [34], was clearly observed in HG-treated cells (Figure 2I). Interestingly, β-asarone ameliorated the HG-induced DNA damage as indicated by reversed 8-OhdG levels and comet tail formation. These results indicate that β-asarone inhibits HG-induced apoptosis and DNA damage in ARPE-19 cells.

### 3.3. β-Asarone Decreases ROS Generation in HG-Treated ARPE-19 Cells

**Figure 3:**
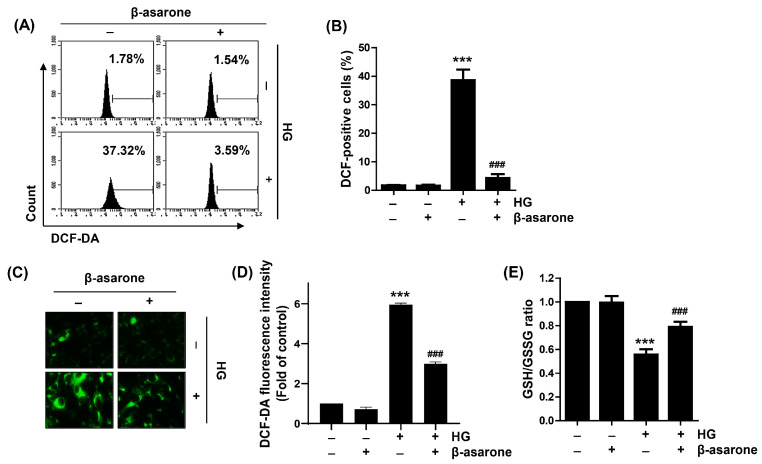
Suppression of HG-induced cytosolic reactive oxygen species (ROS) accumulation by β-asarone in ARPE-19 cells. (**A**–**D**) Cells pre-treated with β-asarone for 1 h were cultured in an HG medium for 1 h and stained with 5,6-carboxy-2′,7′-dichlorodihydrofluorescein diacetate (DCF-DA). (**A**,**B**) Representative images of flow cytometry (**A**) and quantification results (**B**). (**C**) Representative images (green) of cytosolic ROS levels visualized using fluorescence microscopy (200×). (**E**) Reduced glutathione/oxidized glutathione (GSH/GSSG) ratio was measured using a commercially available kit. (**B**,**D**,**E**) Numerical values are represented as the mean ± SD of three independent experiments (*** *p* < 0.001 vs. control group; ### *p* < 0.001 vs. HG-treated group).

As decreased cell viability and the induction of cytotoxicity by HG stimulation were blocked not only by β-asarone but also by the ROS scavenger NAC, we investigated whether the beneficial effects of β-asarone on HG were related to the mitigation of oxidative stress in ARPE-19 cells. In both flow cytometry and fluorescence microscopy results using DCF-DA staining, which reflects the amount of cytosolic ROS generation, increased ROS levels were detected in cells cultured under HG conditions; however, pre-treatment with β-asarone reduced the ROS levels by >90% compared to HG conditions (Figure 3A–D). In addition, as shown in Figure 3E, the GSH/GSSG ratio was significantly reduced when cells were challenged with HG, but not when cells were treated with β-asarone alone. However, β-asarone prevented GSH depletion, indicating that β-asarone alleviates oxidative stress in ARPE-19 cells exposed to HG.

### 3.4. β-Asarone Attenuates HG-induced Production of Mitochondrial Superoxide Radicals and Inactivation of MnSOD in ARPE-19 Cells

**Figure 4:**
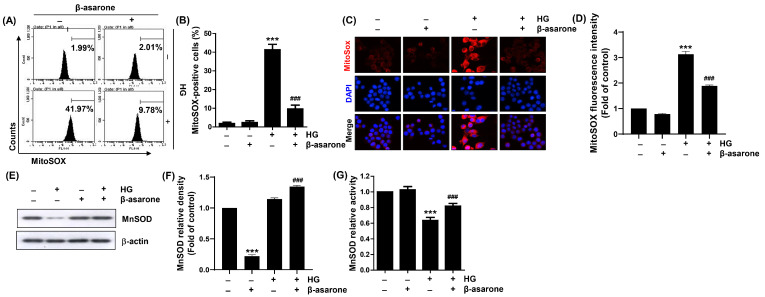
Blockade of HG-induced mitochondrial ROS accumulation by β-asarone in ARPE-19 cells. Cells pre-treated with β-asarone for 1 h were cultured in an HG medium for 1 h and stained with MitoSOX. (**A**,**B**) Representative images of flow cytometry (**A**) and quantification results (**B**). (**C**,**D**) Representative images of mitochondrial ROS levels (red) visualized using fluorescence microscopy (400×). Nuclei (blue) were stained with DAPI. (**E**,**F**) After the lysis of collected cells, equal amounts of protein from each cell lysate were loaded and blotted with manganese superoxide dismutase (MnSOD) and β-actin antibodies. (**G**) Activity of Mn-SOD was measured using a commercially available kit. (**B**,**D**,**F**,**G**) Numerical values are represented as the mean ± SD of three independent experiments (*** *p* < 0.001 vs. control group; ### *p* < 0.001 vs. HG-treated group).

To investigate that mitochondria are the main source of ROS production in HG-treated ARPE-19 cells, mitochondrial superoxide levels were quantified using MitoSOX staining. Similar to the DCF-DA results, the levels of mitochondrial superoxide greatly increased in cells cultured under HG conditions, which was further confirmed by the increased MitoSOX fluorescence intensity observed using fluorescence microscopy (Figure 4A–D). However, β-asarone dramatically abrogated the HG-induced superoxide production. In addition, the expression and activity of MnSOD, a scavenger of mitochondrial superoxide radicals [35,36], were decreased by HG stimulation but were significantly restored in the presence of β-asarone (Figure 4E–G). These results suggest that β-asarone relieved oxidative stress by blocking the production of superoxide, a major ROS produced in the mitochondria, under HG conditions.

### 3.5. β-Asarone Alleviates HG-Induced Inflammatory Response in ARPE-19 Cells

**Figure 5:**
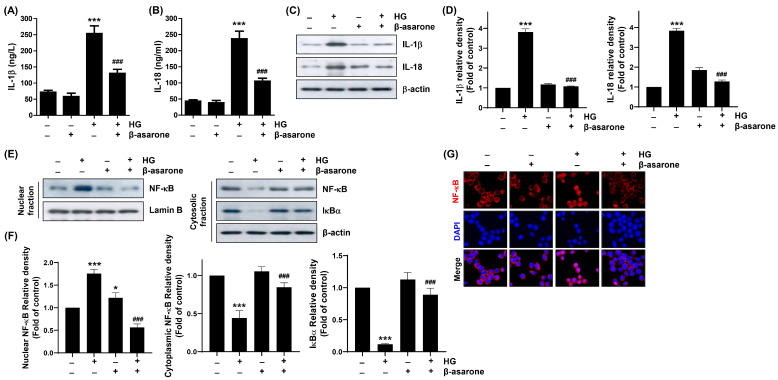
Attenuation of HG-induced cytokine production and nuclear factor-kappa B (NF-κB) activation by β-asarone in ARPE-19 cells. (**A**,**B**) Concentrations of interleukin (IL)-1β (**A**) and IL-18 (**B**) released in the supernatant of cells exposed to HG for 48 h in the presence or absence of β-asarone were measured using enzyme-linked immunosorbent assay (ELISA) kits. (**C**,**D**) Expression levels of IL-1β and IL-18 proteins were determined via Western blot analysis using cells cultured under the same conditions. (**E**–**G**) Cells pre-treated with β-asarone for 1 h were further cultured in an HG medium for another 1 h. (**E**,**F**) Nuclear and cytosolic fractions were used to detect the indicated proteins. (**G**) Cellular localization of p-NF-κB p65 (red) in cells cultured under the same experimental setting was observed using the immunofluorescence assay. Nuclei (blue) were counterstained with DAPI. (**A**,**B**,**D**,**F**) Numerical values are represented as the mean ± SD of three independent experiments (* *p* < 0.05, *** *p* < 0.001 vs. control group; ### *p* < 0.001 vs. HG-treated group).

Next, we investigated the secretion of pro-inflammatory cytokines, such as IL-1β and IL-18, in the supernatants of ARPE-19 cells cultured under HG conditions in the presence or absence of β-asarone to explore the blocking effect of β-asarone on HG-induced inflammatory response. ELISA revealed that HG stimulation increased the inflammatory response as indicated by the enhanced levels of IL-1β and IL-18 in the cell supernatants (Figure 5A,B). Additionally, the protein expression levels of IL-1β and IL-18 were elevated under HG conditions (Figure 5C,D). However, after β-asarone intervention, the levels and protein expression of these inflammatory factors were significantly decreased compared to those in the HG group (Figure 5A–D), indicating that β-asarone counteracts HG-induced inflammation in ARPE-19 cells.

### 3.6. β-Asarone Mitigates NF-κB Signaling Activation in HG-Treated ARPE-19 Cells

As NF-κB plays a critical role in the transcription of inflammation-related factors [37,38], we verified whether the blockade of HG-induced inflammation by β-asarone is dependent on the NF-κB signaling pathway. According to the results of immunoblotting and immunofluorescence assays presented in Figure 5E–G, NF-κB protein levels were significantly increased in the nuclear fraction rather than in the cytoplasmic fraction of cells cultured under HG conditions. Moreover, p-NF-κB was preferentially expressed in the nuclei of HG-treated cells but not in cells treated with β-asarone alone. Additionally, the expression levels of IκB-α were downregulated in the cytoplasm. However, in the presence of β-asarone, the nuclear localization of HG-induced NF-κB as well as the expression levels of p-IκB-α and IκB-α were reduced to the control levels. These results indicate that β-asarone alleviates HG-induced NF-κB activation, thereby reducing the associated inflammatory response.

### 3.7. β-Asarone Attenuates HG-Induced NLRP3 Inflammasome Activation in ARPE-19 Cells

**Figure 6:**
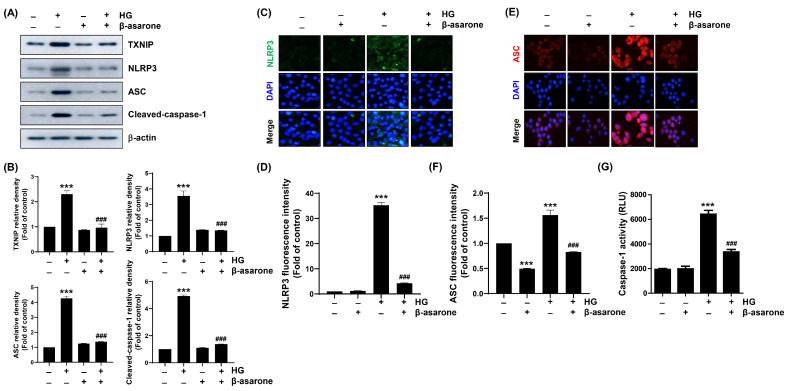
Suppression of HG-induced NOD-like receptor family pyrin domain-containing 3 (NLRP3) inflammasome activation by β-asarone in ARPE-19 cells. Cells pre-treated with β-asarone for 1 h were further cultured in an HG medium for 48 h, and Western blot analysis was conducted to determine the expression levels of the indicated proteins (**A**,**B**). (**C**–**F**) Cellular localization of NLRP3 (green) and apoptosis-associated speck-like protein containing a caspase-recruitment domain (ASC) (red) in cells cultured under same experimental setting was observed using the immunofluorescence assay. Nuclei (blue) were counterstained with DAPI. (**G**) Activity of caspase-1 was measured using a fluorescence assay kit. (**B**,**D**,**F**,**G**) Numerical values are represented as the mean ± SD of three independent experiments (*** *p* < 0.001 vs. control group; ### *p* < 0.001 vs. HG-treated group).

NF-κB-induced NLRP3 inflammasome activation plays critical roles in the onset and progression of DR. NLRP3 inflammasome is a multiprotein complex that mediates the secretion of pro-inflammatory cytokines, including IL-1β and IL-18 [15,17]. As β-asarone attenuated HG-induced secretion of IL-1β and IL-18 and NF-κB activation, we further investigated whether these inhibitory effects contributed to the blocking of the NLRP3 inflammasome pathway. Immunoblotting revealed that the expression levels of thioredoxin (Trx)-interacting protein (TXNIP), an NLRP3 interacting molecule [9,11], and the NLRP3 inflammasome multiprotein complex molecules, ASC and caspase-1, were upregulated under HG conditions, whereas β-asarone reduced these levels similar to those in the control group (Figure 6A,B). To confirm these results, fluorescence and immunofluorescence assays were conducted to measure the intracellular caspase-1 activity and levels of NLRP3 and ASC. We found that caspase-1 activity and NLRP3 and ASC expression levels were notably enhanced under HG conditions but reduced by β-asarone to levels similar to those in the control (Figure 6C–G). These results suggest that β-asarone counteracts the activation of NLRP3 inflammasome under HG conditions in ARPE-19 cells.

## 4. Discussion

Elevated glucose levels are a potent pathogenic feature of DR [39,40]. Excess ROS production due to hyperglycemia initiates oxidative stress and inflammatory responses, which are mechanisms involved in the pathogenesis of DR [3,7]. Antioxidants with anti-inflammatory effects exert inhibitory effects on the onset and progression of DR [41,42,43]. β-asarone, a type of volatile oil, exerts multiple pharmacological effects, including antioxidant and anti-inflammatory effects [19,20,44]. In addition to these pharmacological effects, β-asarone is also known for toxicological effects in hepatoma, mutagenic, and genotoxic action [20]. Even with toxicological effects of β-asarone, it demonstrates therapeutic properties by targeting various pharmacological molecular targets such as apoptotic pathway modulator [20]. In particular, the antioxidant activity of β-asarone is related to the quenching of ROS generation in various experimental systems, including Alzheimer’s disease and diabetic encephalopathy models [23,45,46]. Chang and Teng [47] reported that β-asarone inhibits the production of inflammatory cytokines, including tumor necrosis factor-α (TNF-α), IL-1β, and IL-6, and induction of autophagy, while reducing apoptosis. The results of this study provide the first evidence that β-asarone suppresses NLRP3 inflammasome activation and consequent DNA damage and lytic cell death by reducing ROS production in RPE ARPE-19 cells under HG conditions.

In this study, ARPE-19 cells were treated with HG (20 mM) to mimic hyperglycemia and the ROS levels were measured. Both cytosolic and mitochondrial ROS levels were markedly increased in ARPE-19 cells, confirming that the hyperglycemic state was characterized by high oxidative stress. Similar to previous studies, the present results showed that culturing ARPE-19 cells with HG leads to apoptosis and DNA damage via ROS generation [48,49,50,51]. However, in the presence of β-asarone, the production of ROS and induction of apoptosis and DNA damage were significantly abolished in HG-treated ARPE-19 cells. In particular, the expression and activity of MnSOD, which is an NF-κB-dependent antioxidant that plays a key role in scavenging mitochondrial superoxide [34,35], were decreased under HG conditions but recovered by β-asarone. This result implies that mitochondria are sources of ROS under HG conditions and potentially MnSOD may partially contribute to reduction in oxidative stress by β-asarone. In addition, β-asarone blocked the activation of NF-κB by HG and decreased the production and expression of IL-1β and IL-18, which are pro-inflammatory cytokines associated with NLRP3 inflammasome activation [15,16]. β-asarone inhibits HG-induced NLRP3 inflammasome activation in a myocardial I/R model [23]. These results imply that β-asarone protects ARPE-19 cells from HG-mediated oxidative stress, inflammation, and apoptosis via the regulation of NF-κB-related pathways.

NLRP3 inflammasome plays an important role in the pathogenesis of diabetes and its representative complication, DR [10,11,52]. Singh et al. [53] showed that the blockade of TXNIP activity, which is strongly induced by diabetes and inhibits the antioxidant function of Trx, prevents or slows down the progression of DR by normalizing mitophagic flux and NLRP3 inflammasome activation. This suggests the involvement of the TXNIP–Trx redox pathway in the dysfunction of RPE cells in retinal neurodegenerative diseases, such as DR [54]. Unlike apoptosis, lytic cell death is a programmed inflammatory cell death induced by the activation of caspase-1, which plays a key role in the release of IL-1β and IL-18 [12,52]. NF-κB priming signals for the transcriptional activation of NLRP3, pro-IL-1β, and pro-IL-18 are required for the activation of the NLRP3 inflammasome. Caspase-1 activation following the assembly of the NLRP3 inflammasome complex leads to the cleavage of pro-IL-1β and pro-IL-18 to IL-1β and IL-18, respectively, and the induction of lytic cell death [15,17]. Gan et al. [55] reported that retinal pericytes exposed to hyperglycemic conditions are lost via NLRP3–caspase-1-mediated lytic cell death and that lytic cell death by NLRP3 inflammasome activation is a key process in triggering DR pathogenesis [10,11,52]. The results of this study indicate that β-asarone attenuates HG-stimulated NLRP3 inflammasome-mediated lytic cell death and inactivates NF-κB in ARPE-19 cells, indicating its potential to treat hyperglycemia-induced DR.

**Figure 7:**
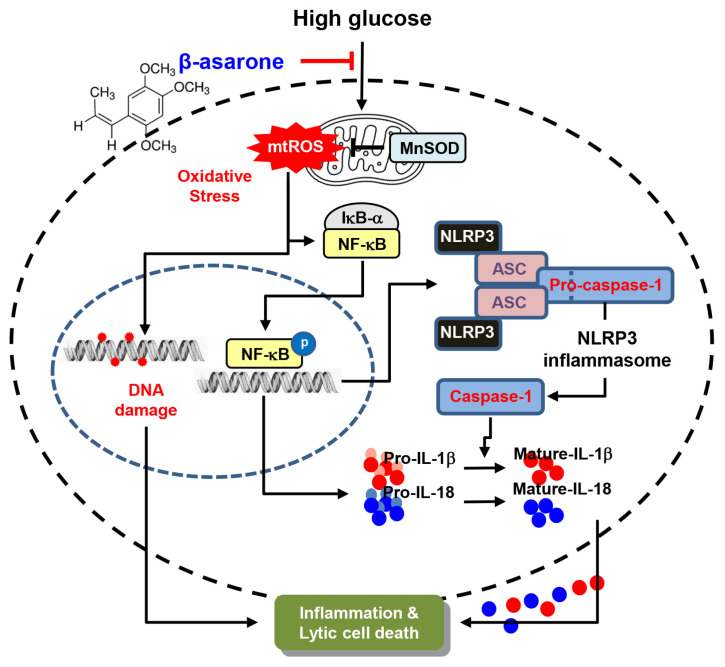
Schematic diagram of the inhibitory effects of β-asarone on mitochondrial ROS-mediated activation of NF-κB/NLRP3 signaling, resulting in the alleviation of inflammation in hyperglycemic RPE cells.

Taken together, our results revealed that β-asarone inhibits the inflammatory response and oxidative stress, the two major steps in the vicious cycle of DR. Moreover, the attenuation of lytic cell death by β-asarone in HG-treated RPE ARPE-19 cells may be due to the inactivation of NF-κB and NLRP3 inflammasomes following oxidative stress relaxation (Figure 7). These results indicate that β-asarone exhibits therapeutic potential for hyperglycemia-induced DR. However, the possible involvement of other pathways in the inhibition of NF-κB and NLRP3 inflammasome requires further evaluation via animal experiments.

## 5. Conclusions

In conclusion, this study demonstrated that β-asarone counteracts HG-induced lytic cell death and DNA damage by reducing the oxidative stress and inflammatory responses in RPE cells. These protective effects may be attributed to its ability to suppress NLRP3 inflammasome-mediated lytic cell death. Our findings suggest β-asarone as a promising preventive and therapeutic agent for hyperglycemia-induced DR.

## Data Availability

All of the data is contained within the article.
